# Comparison of Fine Needle Aspiration and Fine Needle Nonaspiration Cytology of Thyroid Nodules: A Meta-Analysis

**DOI:** 10.1155/2015/796120

**Published:** 2015-09-29

**Authors:** Hongming Song, Chuankui Wei, Dengfeng Li, Kaiyao Hua, Jialu Song, Niraj Maskey, Lin Fang

**Affiliations:** ^1^Department of Breast and Thyroid Surgery, Shanghai Tenth People's Hospital, School of Medicine, Tongji University, Shanghai 200072, China; ^2^Department of General Surgery, Affiliated Hospital of Taishan Medical University, Taian 271000, China

## Abstract

*Background*. Fine needle aspiration cytology (FNAC) and fine needle nonaspiration cytology (FNNAC) are useful cost-effective techniques for preoperatively assessing thyroid lesions. Both techniques have advantages and disadvantages, and there is controversy over which method is superior. This meta-analysis was performed to evaluate the differences between FNAC and FNNAC for diagnosis of thyroid nodules. *Methods*. Primary publications were independently collected by two reviewers from PubMed, Web of Science, Google Scholar, EBSCO, OALib, and the Cochrane Library databases. The following search terms were used: fine needle, aspiration, capillary, nonaspiration, sampling without aspiration, thyroid, and cytology. The last search was performed on February 1, 2015. *Results*. Sixteen studies comprising 1,842 patients and 2,221 samples were included in this study. No statistically significant difference was observed between FNAC and FNNAC groups with respect to diagnostically inadequate smears, diagnostically superior smears, diagnostic performance (accuracy, sensitivity, specificity, negative predictive value, and positive predictive value), area under the summary receiver operating characteristic curve, average score of each parameter (background blood or clot, amount of cellular material, degree of cellular degeneration, degree of cellular trauma, and retention of appropriate architecture), and total score of five parameters. *Conclusion*. FNAC and FNNAC are equally useful in assessing thyroid nodules.

## 1. Introduction

Thyroid nodules are a common clinical problem, and 1–10% are malignant [1]. The incidence of thyroid cancer nearly tripled from 1975 to 2009, primarily as a result of an increase in papillary thyroid carcinoma [2]. Therefore, early diagnosis and treatment have become increasingly important in curing malignant thyroid carcinoma.

Fine needle aspiration cytology (FNAC) has been routinely used as the baseline investigation for diagnosis of nodular thyroid disease. Its advantages include minimal invasion and high sensitivity, specificity, and accuracy [3]. However, it has also disadvantages; the bloody smears caused by negative pressure during aspiration are detrimental to both cell concentration and cell morphology of the specimen, leading to an unsatisfactory specimen and improper cytological interpretation [4–6].

In an attempt to overcome these problems, fine needle nonaspiration cytology (FNNAC) was developed in France in 1982 by Briffod et al. [7] and described by Santos and Leiman in 1988 [6]. FNNAC avoids active aspiration and relies on capillary tension to suck the tissue sample into the needle bore; this reduces bleeding and minimizes trauma to thyroid tissue [8, 9].

There are many conflicting studies regarding the superiority of FNNAC to FNAC [10–18]. Some studies have reported that FNNAC reduced bleeding and obtained higher quality samples [11–13]; other reports have indicated that the diagnostic adequacy of FNAC was higher than FNNAC [17, 18] or that both methods were equally efficient [10, 14, 15]. Studies on the accuracy, sensitivity, specificity, negative predictive value (NPV), and positive predictive value (PPV) of both techniques based on histopathology have also been inconclusive [10, 15, 19–21].

Hence, we have conducted a systematic review and meta-analysis to evaluate the performance of FNAC and FNNAC in diagnosing nodular thyroid disease. We also aim to clarify the diagnostic performance of both techniques, which will provide physicians with a theoretical reference and guidelines to properly select between these two techniques.

## 2. Methods

### 2.1. Electronic Library Search

Relevant publications were collected from PubMed, EBSCO, Google Scholar, OALib, and Cochrane databases. The search keywords used were fine needle, aspiration, capillary, nonaspiration, sampling without aspiration, thyroid, and cytology. There was no restriction on the publication date or language. We removed duplicated publications that were identified in multiple databases.

### 2.2. Study Inclusion/Exclusion Criteria

All relevant titles, abstracts, and full papers identified by the prespecified search strategy were independently screened by two authors (Hongming Song and Chuankui Wei), and irrelevant articles were excluded. Search results were compared, and disagreements were resolved by discussion with the third reviewer (Kaiyao Hua).

The included studies reported comparison of performance between FNAC and FNNAC. Studies that did not refer to thyroid nodules and those that did not compare the cytological findings with histological results were excluded from this study. Letters, reviews, abstracts, editorial materials, and animal trials were also excluded from this study.

### 2.3. Assessment of Smear Quality

The scoring system invented by Mair et al. has been widely used to compare the smear quality obtained by FNAC and FNNAC in numerous studies [22], including breast lesions [18], thyroid nodules [10, 15, 19–21], lymph nodes, pancreatic masses, and liver lesions [17, 29]. This scoring system consists of five objective parameters: (1) background blood or clot, (2) amount of cellular material, (3) degree of cellular degeneration, (4) degree of cellular trauma, and (5) retention of appropriate architecture (Table 1). In this review, the quality of smears obtained by both techniques was scored according to Mair et al. scoring system [22]. A cumulative score ranging between 0 and 10 points was calculated for each smear and then categorized into the following three categories:

- Category 1 (scores 0–2): smear unsuitable for diagnosis.
- Category 2 (scores 3–6): smear adequate for cytological diagnosis.
- Category 3 (scores 7–10): diagnostically superior smear [25].

We also calculated the diagnostic performance of FNAC and FNNAC by comparing the cytological diagnosis of thyroid nodules with the histological results, regardless of whether the included studies adopted Mair et al. scoring system.

### 2.4. Data Extraction

We extracted the following data from the included studies: the number of Categories 1 and 3 smears, the average score (mean ± SD) of each of the five objective parameters (background blood or clot, amount of cellular material, degree of cellular degeneration, degree of cellular trauma, and retention of appropriate architecture), and the average total score of the five parameters (mean ± SD). The numbers of true positive, false positive, false negative, and true negative results were evaluated. The diagnostic performance (accuracy, sensitivity, specificity, NPV, and PPV) of both techniques was extracted. The name of the first author, year of publication, study design, number of patients, number of lesions, and needle gauge were also reviewed and recorded (Table 2).

### 2.5. Assessing the Risk of Bias

Risk of bias was independently assessed by the two main authors using Review Manager software (RevMan, version 5.3, Copenhagen, The Nordic Cochrane Centre, The Cochrane Collaboration, 2014) to evaluate the methodological quality of all included studies. The following six aspects were evaluated: random sequence generation, allocation concealment, blinding, incomplete outcome data, selective reporting, and other bias. All studies were classified as “unclear,” “yes,” or “no” to indicate “uncertain bias,” “low-risk bias,” or “high-risk bias,” respectively. The assessment of risk of bias is described in Table 3.

### 2.6. Statistical Analysis

The data from included studies were analyzed using Review Manager software (RevMan, version 5.3, Copenhagen, The Nordic Cochrane Centre, The Cochrane Collaboration, 2014). Each study was weighted by its sample size. For dichotomous variables such as the smear quality and accuracy of FNAC and FNNAC, odds ratios (OR) and 95% confidence intervals (95% CI) were calculated. The weighted mean difference and standardized mean difference were computed for continuous variables that had the same or different units in the assessing system, respectively. The mean difference (MD) and 95% CI were computed for the average score of each parameter and the average total score of the five parameters. Heterogeneity among the studies was assessed using the *χ* ^2^ test and *I* ^2^ statistics. If the heterogeneity test did not reveal statistical significance (*I* ^2^ < 50%, *P* > 0.1), the fixed-effects model was adopted; otherwise, the random-effects model was used. If the *P* value was less than 0.05 and 95% CI did not contain the value 1 for OR or the value 0 for MD, the OR and MD were considered to be statistically significant. Publication bias was assessed by the funnel plot. The sensitivity analysis of the results was performed using the leave-one-out approach. The summary receiver operating characteristic (SROC) curve analysis was performed using Meta-Disc version 1.4 software. The corresponding area under the curve (AUC) was calculated as a global measurement of test performance; the closer the AUC to 1, the better the test performance.

## 3. Results

### 3.1. Search Results

**Figure 1:**
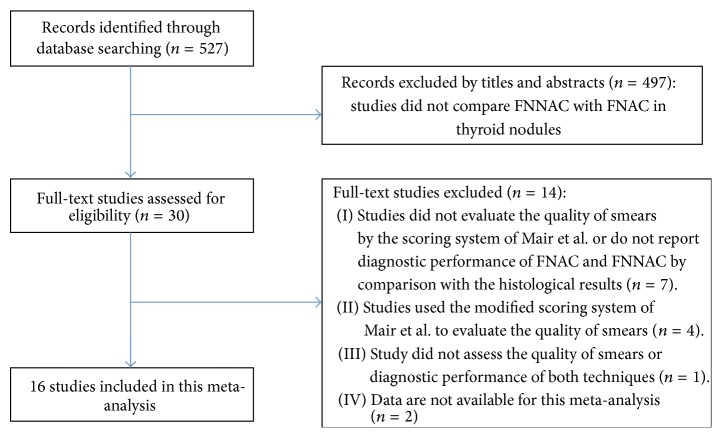
Flow chart of selection of eligible studies.

A total of 527 records were identified from the databases. Among them, 30 full-text articles were assessed for potential eligibility. Seven articles were excluded because they did not use the Mair et al. scoring system or did not report the diagnostic performance of FNAC and FNNAC [3–6, 9, 30, 31]. Four articles that used the modified scoring system of Mair et al. were excluded (1–3 parameters were excluded from the Mair et al. scoring system) [16, 32–34]. One article was excluded owing to lack of assessment of smear quality and the diagnostic performance of FNAC and FNNAC [35]. Two articles that did not have available data for meta-analysis were excluded [36, 37]. A final total of 16 articles met the inclusion criteria [8, 10–15, 19–21, 23–28]. The steps taken in selecting eligible articles are shown in Figure 1.

### 3.2. Characteristics of the Included Studies

In this meta-analysis, the 16 included studies involved 1,842 individual patients and 2,221 samples collected by FNAC and FNNAC. Of these studies, 15 were prospective and only one was retrospective in design. The studies have great differences in the number of patients and samples, needle gauge, sex ratio, and mean age of patients. The results included diagnostically inadequate and superior smears, diagnostic performance (accuracy, sensitivity, specificity, NPV, and PPV), average scores of each parameter, and average total scores of the five parameters of the Mair et al. scoring system. Diagnostically inadequate smears collected using both techniques were reported in 12 studies [8, 10–14, 20, 23–27], while superior smears collected using both techniques were reported in 11 studies [8, 10–14, 23–27]. The accuracy of both techniques as confirmed by histopathology was assessed in five studies [10, 15, 19–21]; among these, sensitivity, specificity, NPV, and PPV were extracted from four studies [10, 15, 19, 21], the average score of each of the five parameters was measured for* both techniques* in five studies [8, 10, 13, 24, 28], and the average of the total scores was calculated in five studies [8, 10, 13, 14, 24]. The characteristics of the included studies are described in Table 2.

### 3.3. The Primary Meta-Analysis Results

#### 3.3.1. Comparison of the Quality of Smears Collected by FNAC versus FNNAC

**Figure 2:**
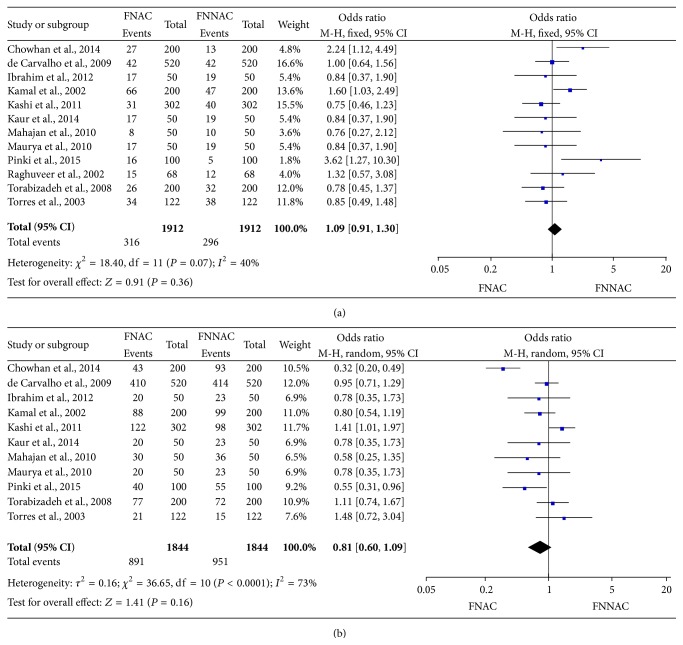
Forest plots showing the quality of specimens obtained by FNAC and FNNAC. (a) Unsuitable for diagnosis, (b) diagnostically superior.

The number of diagnostically superior smears collected via FNAC compared with FNNAC was assessed in 11 studies [8, 10–14, 23–27]. The proportion of diagnostically superior smears in the FNAC and FNNAC groups ranged from 14.6 to 78.8% and from 12.3 to 79.6% in 11 studies, respectively. Smears unsuitable for diagnosis were collected using both techniques in 12 studies [8, 10–14, 20, 23–27]. The proportion of smears unsuitable for diagnosis ranged from 8.1 to 34.0% and from 8.1 to 38.0% in the FNAC and FNNAC groups, respectively. The pooled proportion of diagnostically superior smears were 891/1,844 (48.3%) and 951/1,844 (51.6%) in the FNAC and FNNAC groups, respectively; there was no statistically significant difference between the groups (MD 0.81, 95% CI 0.60–1.09, and *P* = 0.16) (Figure 2(b)). Similarly, the pooled proportion of smears unsuitable for diagnosis was 316/1,912 (16.5%) and 296/1,912 (15.5%) in the FNAC and FNNAC groups, respectively; no statistically significant difference was observed between the groups (MD 1.09, 95% CI 0.91–1.30, and *P* = 0.36) (Figure 2(a)).

#### 3.3.2. Comparison of the Diagnostic Performance of Both Techniques

**Figure 3:**
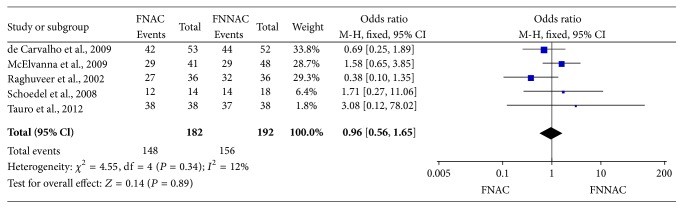
Forest plot showing the diagnostic accuracy of FNNAC and FNAC techniques.

**Figure 4:**
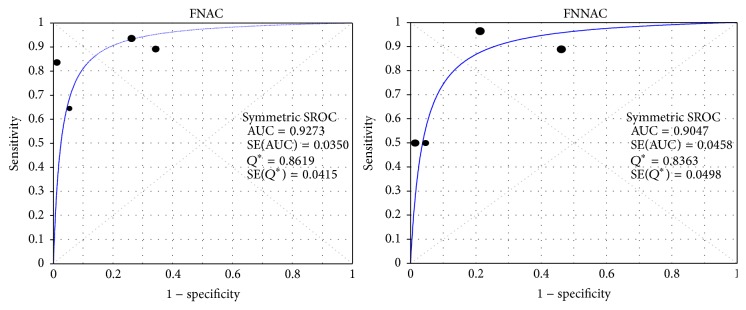
The summary receiver operating characteristic (SROC) curves for FNAC and FNNAC. The areas under the curve (AUCs) for FNAC and FNNAC were 0.9273 ± 0.0350 and 0.9047 ± 0.0458, respectively. There was no significant difference between the AUCs for FNAC and FNNAC (*P* > 0.05). Symmetric SROC curve fitted using Moses constant of linear model. SE: standard error. AUC: area under the curve.

A complete histopathological analysis is essential to make a definite diagnosis for thyroid lesions. Cytological findings obtained by FNAC and FNNAC were confirmed by histopathological analysis in five studies [10, 15, 19–21]. The respective pooled accuracy of FNAC and FNNAC was 148/182 (81.32%) and 156/192 (81.25%); there was no statistically significant difference in diagnostic accuracy between FNAC and FNNAC (MD 0.96, 95% CI 0.56–1.65, and *P* = 0.89) (Figure 3). The sensitivity, specificity, NPV, and PPV were extracted from four studies [10, 15, 19, 21], with no statistically significant difference observed between FNAC and FNNAC (Table 4). To analyze the SROC, the performances of the four diagnostic studies are shown in Table 5. The areas under the SROC curves were 0.9273 ± 0.0350 for FNAC and 0.9047 ± 0.0458 for FNNAC. No significant difference was observed between the AUCs of FNAC and FNNAC (Figure 4).

### 3.4. The Subgroup Analysis of the Mair et al. Scoring System

#### 3.4.1. Mair et al. Scores of FNAC and FNNAC Groups

**Figure 5:**
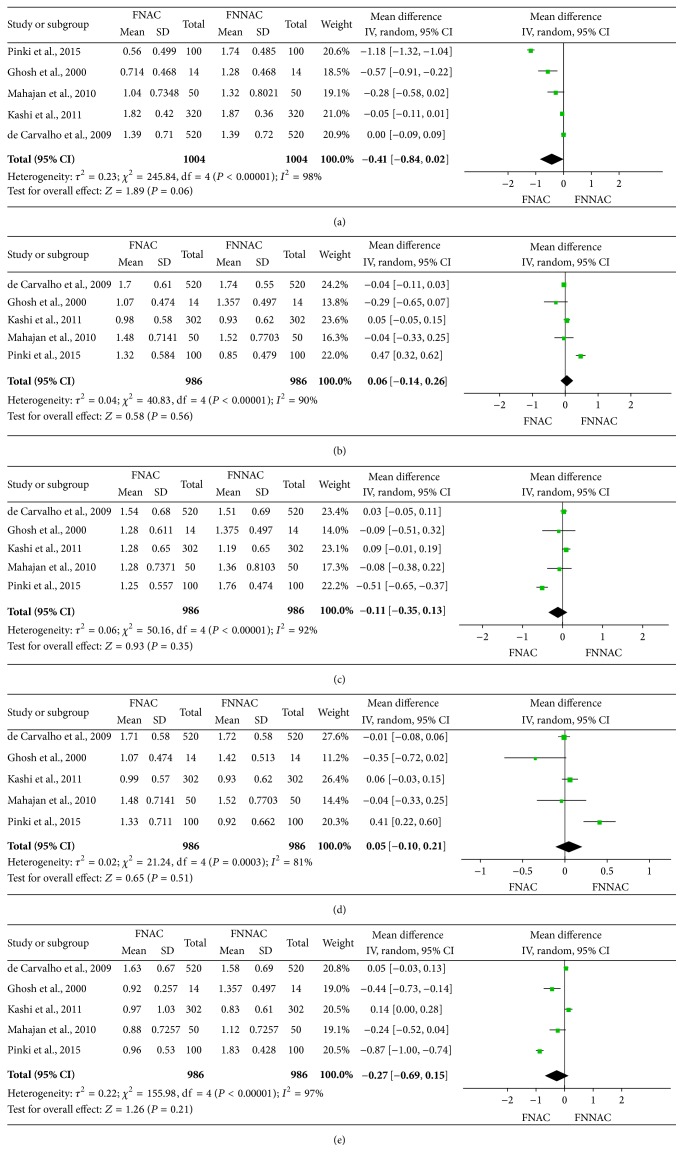
Forest plots showing average scores of the five evaluation parameters for FNNAC and FNAC. (a) Background blood or clot, (b) degree of cellular trauma, (c) amount of cellular material, (d) degree of cellular degeneration, and (e) retention of appropriate architecture.

**Figure 6:**
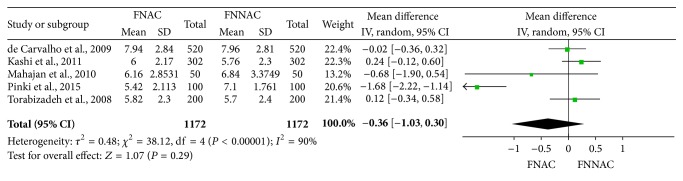
Forest plot showing the mean of the total scores of FNNAC and FNAC techniques.

The average score for each parameter of the samples obtained by FNAC and FNNAC was reported in five included studies [8, 10, 13, 24, 28], and five studies calculated the mean of the total scores of each sample [8, 10, 13, 14, 24]. There was no statistically significant difference in the average scores of the five parameters or the mean of the total scores between the FNAC and FNNAC groups (Table 4). Forest plots show the average scores of the five parameters (Figure 5) and the mean of the total scores (Figure 6) for the FNNAC and FNAC techniques.

## 4. Discussion

Although many studies have compared the efficiency of FNAC and FNNAC techniques in evaluating thyroid nodules, there is no clear agreement as to which method performs better. To the best of our knowledge, this is the first meta-analysis to evaluate the smear quality and diagnostic performance of FNAC and FNNAC. The five parameters used for performance evaluation may interfere with each other; hence, if the scoring system excluding one or more parameters is used, the average score and total score may not accurately reflect each parameter and the smear quality, respectively. Therefore, we strictly selected studies that used the scoring system of Mair et al. to assess the quality of smears obtained by FNAC and FNNAC.

It is well known that the smear quality may affect the cytological diagnosis of thyroid nodules. In this meta-analysis, we compared the quality of smears collected by FNAC and FNNAC using the Mair et al. scoring system and found no statistically significant difference between the quality of smears obtained by FNNAC and FNAC. A larger number of smears collected by FNNAC tended to be superior smears compared with those collected by FNAC; however, this was not statistically significant. We also observed a similar rate of smears unsuitable for diagnosis between FNNAC and FNAC groups.

“Background blood or clot” and “amount of cellular material” are two important criteria in assessing the quality of smears [1]. In theory, FNAC may cause more hemorrhage than FNNAC, and FNNAC may produce better cellular material than FNAC. Considering that the thyroid is a vascular organ, hemorrhage is also an important factor that can seriously affect the interpretation of results and thus lead to inaccurate diagnosis. In this meta-analysis, we did not find any difference in the background blood or clot, amount of cellular material, degree of cellular degeneration, degree of cellular trauma, retention of appropriate architecture, or mean score of the five parameters between FNNAC and FNAC groups.

The objective of fine needle biopsy is to investigate thyroid nodules. The diagnostic accuracy is important in determining whether patients with suspicious thyroid nodules need surgery. Five included studies reported the diagnostic accuracies of both techniques [10, 15, 19–21]. We compared the diagnosis using both techniques with the histological results and found that the diagnostic accuracy was not significantly different between FNAC and FNNAC. There was also no statistical difference between FNAC and FNNAC regarding sensitivity, specificity, NPV, or PPV of diagnosis [10, 15, 19, 21]. As a global measurement of diagnostic performance in a meta-analysis, the SROC curve summarized the joint distribution of sensitivity and specificity; the AUCs of FNAC and FNNAC were near to 1, with no significant difference observed between them, suggesting that both techniques are useful in diagnosing thyroid nodules.

Some studies reported that the execution order of FNNAC and FNAC techniques plays an important role in affecting the quality of smears. Although the order of FNNAC and FNAC sampling was preplanned in most of the included studies (FNAC followed by FNNAC was performed on patients in group A, FNNAC followed by FNAC was conducted in group B, or the technique used for biopsy was alternated sequentially for each patient), three studies had a high risk of bias based on low-quality data. One study conducted FNAC followed by FNNAC sampling for all cases, and two studies reversed the order of FNNAC and FNAC techniques for all patients. This might have led to the differences in results caused by the order of FNNAC and FNAC sampling. However, when we excluded these three studies, the execution order of FNNAC and FNAC made no difference to the quality of smears.

This meta-analysis had some potential limitations. First, numerous factors may have affected the consistency of results, as the included studies used various fine needle biopsy protocols (such as varying needle gauge and size of syringe volume). Moreover, there were differences in the level of suction pressure applied and the insertion depth of fine needles. These factors might have caused a small but possible risk of bias. Second, the sample size of included studies was small, especially for comparing the diagnostic accuracy of both techniques with the histological results; this might lead to the small-study effect; thus, the results obtained should be considered with caution. Third, we did not assess other complications such as nerve damage, tissue trauma, tumor seeding, or vascular injury associated with both techniques, owing to a lack of data in the included studies. Finally, some studies reported that FNNAC combined with FNAC can obtain better quality cellular material [8, 9], while other studies reported that a better diagnostic accuracy can be achieved by combining both techniques [13, 23, 26]. This suggests that a combination of both techniques may be more suitable for the investigation of patients with thyroid nodules. However, because of a lack of adequate evidence, we could not conduct a meta-analysis to compare the performance of a combination of both techniques with FNNAC or FNAC alone.

## 5. Conclusion

FNNAC and FNAC techniques are equally useful in the assessment of thyroid nodules. The selection of technique may be dependent on the personal preference of the operator.

## Figures and Tables

**Table 1:** The Mair et al. scoring system [22].

Criteria	Quantitative description	Point score
Background blood/clot	Large amount, great compromise of diagnosis	0
	Moderate amount, diagnosis possible	1
	Minimal amount, diagnosis	2

Amount of cellular material	Minimal to absent, diagnosis not possible	0
	Sufficient for cytodiagnosis	1
	Abundant, diagnosis possible	2

Degree of cellular degeneration	Marked, diagnosis impossible	0
	Moderate, diagnosis possible	1
	Minimal, diagnosis easy	2

Degree of cellular trauma	Marked, diagnosis impossible	0
	Moderate, diagnosis possible	1
	Minimal, diagnosis obvious	2

Retention of appropriate architecture	Minimal to absent nondiagnostic	0
	Moderate, some preservation of, for example, follicle, papillae, and acini	1
	Excellent architectural display closely reflecting histology, diagnosis obvious	2

**Table 2:** Characteristics of studies included in this meta-analysis.

First author's name	Year of publication	Number of patients FNAC/FNNAC	Number of nodules FNAC/FNNAC	Histopathological diagnosis FNAC/FNNAC	Study design	Needle gauge	Mair et al. scoring system [22]	Patient's age and gender
de Carvalho [10]	2009	260/260	520/520	58/58	Prospective	23	Yes	238 females, 22 males, and age of 43.2 ± 12.6
Schoedel [21]	2008	122/122	180/180	23/23	Prospective	25	No	The ratio of female to male was about 6 : 1, with average age of 57.3
Raghuveer [20]	2002	68/68	68/68	36/36	Prospective	23 or 24	Yes	—
McElvanna [19]	2009	65/65	65/65	65/65	Retrospective	23	No	58 females, 7 males, and average age of 49
Tauro [15]	2012	50/50	50/50	38/38	Prospective	23	No	47 females, 3 males, and age of 39.16 ± 11.47
Maurya [11]	2010	50/50	50/50	—	Prospective	23	Yes	—
Kamal [12]	2002	200/200	200/200	—	Prospective	23 or 24	Yes	173 females, 27 males
Mahajan and Sharma [8]	2010	50/50	50/50	—	Prospective	—	Yes	—
Pinki [13]	2015	100/100	100/100	—	Prospective	22	Yes	—
Ibrahim [23]	2012	50/50	50/50	—	Prospective	25	Yes	—
Kashi [24]	2011	302/302	302/302	—	Prospective	25	Yes	289 females, 13 males, and age of 43.83 ± 12.9
Chowhan [25]	2014	200/200	200/200	—	Prospective	24	Yes	26 males, 174 females
Torabizadeh [14]	2008	200/200	200/200	—	Prospective	/	Yes	189 females, 11 males, and age of 43.36 ± 12.3
Kaur [26]	2014	50/50	50/50	—	Prospective	23	Yes	—
Torres [27]	2003	61/61	122/122	—	Prospective	22	Yes	61 females (100%), age of 49.2 ± 15.3
Ghosh [28]	2000	14/14	14/14	—	Prospective	23–25	Yes	—

FNAC: fine needle aspiration cytology, FNNAC: fine needle nonaspiration cytology, and —: not available.

**Table 3:** Risk of bias assessment of included studies.

Study	Sequence generation	Allocation concealment	Blinding	Incomplete outcome data	Selective reporting	Other bias
de Carvalho et al. [10]	Low	Low	Low	Low	Low	Low
Schoedel et al. [21]	Low	Low	Low	Low	Low	Low
Raghuveer et al. [20]	High	Low	Low	Low	Low	Low
McElvanna et al. [19]	Low	Low	Low	Low	Low	Low
Tauro et al. [15]	Low	Low	Low	Low	Low	Low
Maurya et al. [11]	Low	Unclear	Low	Low	Low	Low
Kamal et al. [12]	Low	Unclear	Low	Low	Low	Low
Mahajan and Sharma [8]	Low	Unclear	Unclear	Low	Low	Low
Pinki et al. [13]	High	Low	Low	Low	Low	Low
Ibrahim et al. [23]	Low	Low	Low	Low	Low	Low
Kashi et al. [24]	Low	Unclear	Low	Low	Low	Low
Chowhan et al. [25]	High	Low	Low	Low	Low	Low
Torabizadeh et al. [14]	Low	Low	Low	Low	Low	Low
Kaur et al. [26]	Low	Low	Low	Low	Low	Low
Torres et al. [27]	Low	Low	Low	Low	Low	Low
Ghosh et al. [28]	Low	Unclear	Low	Low	Low	Low

**Table 4:** Analysis of the results.

Results	Number of studies	Sample size FNAC/FNNAC	Overall effect size	95% CI	*P* value	Heterogeneity	
						*I* ^2^	*P*
Unsuitable for diagnosis	12 [8, 10–14, 20, 23–27]	1912/1912	OR = 1.09	(0.91, 1.30)	0.36	40%	0.07
Diagnostically superior	11 [8, 10–14, 23–27]	1844/1844	OR = 0.81	(0.60, 1.09)	0.16	73%	<0.0001
Background blood or clot	5 [8, 10, 13, 24, 28]	986/986	MD = −0.41	(−0.84, 0.02)	0.06	98%	<0.0001
Degree of cellular trauma	5 [8, 10, 13, 24, 28]	986/986	MD = 0.06	(−0.14, 0.26)	0.56	90%	<0.00001
Amount of cellular material	5 [8, 10, 13, 24, 28]	986/986	MD = −0.11	(−0.35, 0.13)	0.35	92%	<0.00001
Degree of cellular degeneration	5 [8, 10, 13, 24, 28]	986/986	MD = 0.05	(−0.10, 0.21)	0.51	81%	0.0003
Retention of appropriate architecture	5 [8, 10, 13, 24, 28]	986/986	MD = −0.27	(−0.69, 0.15)	0.21	97%	<0.00001
Total score of five parameters	5 [8, 10, 13, 14, 24]	1172/1172	MD = −0.36	(−1.03, 0.30)	0.29	90%	<0.00001
Accuracy	5 [10, 15, 19–21]	182/192	OR = 0.96	(0.56, 1.65)	0.89	12%	0.34
Sensitivity	4 [10, 15, 19, 21]	146/154	OR = 1.35	(0.35, 5.19)	0.66	0%	0.74
Specificity	4 [10, 15, 19, 21]	146/154	OR = 1.13	(0.56, 2.29)	0.73	20%	0.26
Negative predictive value (NPV)	4 [10, 15, 19, 21]	146/154	OR = 1.20	(0.35, 4.11)	0.78	0%	0.78
Positive predictive value (PPV)	4 [10, 15, 19, 21]	146/154	OR = 1.17	(0.50, 2.76)	0.71	0%	0.46

OR: odds ratios, MD: mean difference.

**Table 5:** Performance of the four diagnostic studies.

Study	FNAC/FNNAC								
	Histopathological diagnosis	TP	FP	FN	TP	Performance in diagnosis		Diagnostic criteria for malignancy	Notes
						Sensitivity (%)	Specificity (%)		
de Carvalho et al. 2009 [10]	53/52	14/13	10/8	1/0	28/31	93.3/100	73.7/79.5	Suspicious or positive	Excluded ND
McElvanna et al. 2009 [19]	41/48	8/8	11/18	1/1	21/21	88.9/88.9	65.6/53.8	Not specified	Excluded inadequate cytology specimens
Schoedel et al. 2008 [21]	14/16	4/4	0/0	2/4	8/10	66.7/50	100/100	Suspicious or positive	Excluded I and ND
Tauro et al. 2012 [15]	38/38	2/1	0/0	0/1	36/36	100/50	100/100	Not specified	

TP: true positive, TN: true negative, FP: false positive, and FN: false negative. Sensitivity (%) = TP/(TP + FN) *∗* 100%; specificity (%) = TN/(TN + FP) *∗* 100%. ND: nondiagnostic and I: indeterminate, including follicular lesion and atypical cells present.

## References

[1] Gharib H. (1994). Fine-needle aspiration biopsy of thyroid nodules: advantages, limitations, and effect. *Mayo Clinic Proceedings*.

[2] Davies L., Welch H. G. (2014). Current thyroid cancer trends in the United States. *JAMA Otolaryngology—Head & Neck Surgery*.

[3] Krishnappa P., Ramakrishnappa S. (2014). Cytological evaluation of thyroid lesions by fine needle aspiration versus non-aspiration cytology techniques—a comparative study. *International Journal of Current Research and Review*.

[4] Romitelli F., Di Stasio E., Santoro C., Iozzino M., Orsini A., Cesareo R. (2009). A comparative study of fine needle aspiration and fine needle non-aspiration biopsy on suspected thyroid nodules. *Endocrine Pathology*.

[5] Ramachandra L., Kudva R., Rao B. H. A., Agrawal S. (2011). A comparative study of fine needle aspiration cytology (FNAC) and fine needle non-aspiration cytology (FNNAC) technique in lesions of thyroid gland. *The Indian Journal of Surgery*.

[6] Santos J. E. C., Leiman G. (1988). Nonaspiration fine needle cytology. Application of a new technique to nodular thyroid disease. *Acta Cytologica*.

[7] Briffod M., Gentile A., Hebert H. (1982). Cytopuncture in the follow-up of breast carcinoma. *Acta Cytologica*.

[8] Mahajan P., Sharma P. R. (2010). Fine-needle aspiration versus non aspiration technique of cytodiagnosis in thyroid lesions. *JK Science*.

[9] Rizvi S. A., Husain M., Khan S., Mohsin M. (2005). A comparative study of fine needle aspiration cytology versus non-aspiration technique in thyroid lesions. *The Surgeon*.

[10] de Carvalho G. A., Paz-Filho G., Cavalcanti T. C., Graf H. (2009). Adequacy and diagnostic accuracy of aspiration vs. capillary fine needle thyroid biopsies. *Endocrine Pathology*.

[11] Maurya A. K., Mehta A., Mani N. S., Nijhawan V. S., Batra R. (2010). Comparison of aspiration vs non-aspiration techniques in fine-needle cytology of thyroid lesions. *Journal of Cytology*.

[12] Kamal M. M., Arjune D. G., Kulkarni H. R. (2002). Comparative study of fine needle aspiration and fine needle capillary sampling of thyroid lesions. *Acta Cytologica*.

[13] Pinki P., Alok D., Ranjan A., Chand M. N. (2015). Fine needle aspiration cytology versus fine needle capillary sampling in cytological diagnosis of thyroid lesions. *Iranian Journal of Pathology*.

[14] Torabizadeh Z., Kashi Z., Naghshvar F., Akhi A., Shahidi M., Khalilian A. (2008). Comparison of the adequacy of specimens provided by fine needle aspiration, fine needle non-aspiration sampling and combined technique in thyroid nodules. *Journal of Mazandaran University of Medical Sciences*.

[15] Tauro L. F., Lobo G. J., Fernandes H. (2012). A comparative study on fine needle aspiration cytology versus fine needle capillary cytology in thyroid nodules. *Oman Medical Journal*.

[16] Zhou J.-Q., Zhang J.-W., Zhan W.-W. (2014). Comparison of fine-needle aspiration and fine-needle capillary sampling of thyroid nodules. *Cancer Cytopathology*.

[17] Bharathi K., Anuratha S., Khalique A., Venkatesh S. (2012). A prospective study to compare the aspiration and non aspiration techniques in fine needle cytology of lymphnode and to evaluate the diagnostic accuracy of aspiration cytology in lymphnode lumps. *International Journal of Biological and Medical Research*.

[18] Bharathi K., Venkatesh S. (2012). Comparison of aspiration vs non aspiration techniques in fine needle cytology of breast lesions. *Journal of Medical Science and Technology*.

[19] McElvanna K., Pyper P. C., Miller K. (2009). A comparison of fine-needle aspiration versus non-aspiration cytology of thyroid nodules. *The Internet Journal of Surgery*.

[20] Raghuveer C. V., Leekha I., Pai M. R., Adhikari P. (2002). Fine needle aspiration cytology versus fine needle sampling without aspiration. A prospective study of 200 cases. *Indian Journal of Medical Sciences*.

[21] Schoedel K. E., Tublin M. E., Pealer K., Ohori N. P. (2008). Ultrasound-guided biopsy of the thyroid: a comparison of technique with respect to diagnostic accuracy. *Diagnostic Cytopathology*.

[22] Mair S., Dunbar F., Becker P. J., Du Plessis W. (1989). Fine needle cytology—is aspiration suction necessary? A study of 100 masses in various sites. *Acta Cytologica*.

[23] Ibrahim A.-M. R., Moawad M. M., Al-Hamead A., Al-Satar A., Shahein M. (2012). Cytological evaluation of thyroid lesions. *Asian Academy of Management Journal*.

[24] Kashi Z., Torabizadeh Z., Akha O., Yaseri A., Shahidi M. H., Mokhtare M. (2011). Combination of aspiration and non-aspiration fine needle biopsy for cytological diagnosis of thyroid nodules. *Caspian Journal of Internal Medicine*.

[25] Chowhan A. K., Babu K. S., Sachan A. (2014). Should we apply suction during fine needle cytology of thyroid lesions? A prospective study of 200 cases. *Journal of Clinical and Diagnostic Research*.

[26] Kaur D. S., Garg D. U., Kaur D. S., Singh D. K., Kamra D., Verma D. S. (2014). Comparison of aspiration VS non-aspiration techniques in fine-needle cytology of thyroid lesions. *IOSR Journal of Dental and Medical Sciences*.

[27] Torres M. R., Rosas R. J., Leon Jr E. P. (2003). Punção de tireóide: valor da associação de duas técnicas. *Arquivos Brasileiros de Endocrinologia & Metabologia*.

[28] Ghosh A., Misra R. K., Sharma S. P., Singh H. N., Chaturvedi A. K. (2000). Aspiration vs nonapiration technique of cytodiagnosis—a critical evaluation in 160 cases. *Indian Journal of Pathology and Microbiology*.

[29] Storch I. M., Sussman D. A., Jorda M., Ribeiro A. (2007). Evaluation of fine needle aspiration vs. fine needle capillary sampling on specimen quality and diagnostic accuracy in endoscopic ultrasound-guided biopsy. *Acta Cytologica*.

[30] Braun H., Walch C., Beham A., Moinfar F. (1997). Fine needle capillary cytology versus fine needle aspiration cytology—a comparison of quality between puncture techniques in the ENT area. *Laryngo-Rhino-Otologie*.

[31] Tublin M. E., Martin J. A., Rollin L. J., Pealer K., Kurs-Lasky M., Ohori N. P. (2007). Ultrasound-guided fine-needle aspiration versus fine-needle capillary sampling biopsy of thyroid nodules: does technique matter?. *Journal of Ultrasound in Medicine*.

[32] Haddadi-Nezhad S., Larijani B., Tavangar S. M., Nouraei S. M. (2003). Comparison of fine-needle-nonaspiration with fine-needle-aspiration technique in the cytologic studies of thyroid nodules. *Endocrine Pathology*.

[33] Malik N. P., Jain M., Sharma C. V. (2013). Comparison of aspiration versus non-aspiration technique of cytodiagnosis in thyroid lesions. *Indian Academy of Clinical Medicine*.

[34] Dey P., Ray R. (1993). Comparison of fine needle sampling by capillary action and fine needle aspiration. *Cytopathology*.

[35] Nyonyintono J., Fualal J., Wamala D., Galukande M. (2011). Comparing aspiration and non-aspiration fine needle techniques in cytodiagnosis of thyroid nodules. *East and Central African Journal of Surgery*.

[36] Srikanth S., Anandam G., Kashif M. M. (2014). A comparative study of fine-needle aspiration and fine-needle non-aspiration techniques in head and neck swellings. *Indian Journal of Cancer*.

[37] Hamaker R. A., Moriarty A. T., Hamaker R. C. (1995). Fine-needle biopsy techniques of aspiration versus capillary in head and neck masses. *The Laryngoscope*.

